# Significance of p16 in Site-Specific Human Papillomavirus-Positive and Negative Head and Neck Squamous Cell Carcinoma (HNSCC)

**DOI:** 10.7759/cureus.63594

**Published:** 2024-07-01

**Authors:** Vemparala Priyatha, Hemaakshi Gupta, Pavani Narsapuram, Fatima Ahmed, Haitham Alhussain, Ania Arfa, Mohammed Taha Hussain, Adnan Abdelrahman Abyad Eltayeb, Mohd Abdul Mateen, Priyadarshi Prajjwal

**Affiliations:** 1 Internal Medicine, All India Institute of Medical Sciences, Bhubaneswar, Bhubaneswar, IND; 2 Medicine, American University of the Caribbean, Cupecoy, SXM; 3 Medicine, Kamineni Institute of Medical Sciences, Narketpally, IND; 4 Internal Medicine, Bahria University Medical and Dental College, Karachi, PAK; 5 Epidemiology and Public Health, King Fahad Hospital Hofuf, Ministry of Health, Al-Ahsa, SAU; 6 Medicine, Shadan Institute of Medical Sciences Teaching Hospital and Research Centre, Hyderabad, IND; 7 Medicine, Bashaier Hospital, Khartoum, SDN; 8 Internal Medicine, Shadan Institute of Medical Sciences Teaching Hospital and Research Centre, Hyderabad, IND; 9 Neurology, Bharati Vidyapeeth University Medical College, Pune, IND

**Keywords:** carcinoma, neck, prognostic biomarker, p16, hpv, hnscc

## Abstract

Background: Head and neck squamous cell carcinoma (HNSCC) represents a group of cancers characterized by diverse origins and changing epidemiological patterns. The significance of high-risk human papillomavirus (HPV) infection in certain HNSCC cases has gained attention for its impact on the disease's behavior. Our current research focused on exploring the importance of using p16 as an HNSCC biomarker, particularly in the context of HPV infection, assessing its value in prognosis, and examining its variation across different tumor locations.

Materials and methods: A retrospective analysis was carried out on 100 HNSCC patients from a tertiary care center, with particular attention paid to p16 expression, HPV status, clinic-pathological characteristics, and prognosis. HPV was detected using polymerase chain reaction (PCR) techniques, and p16 expression was evaluated by immunohistochemistry. According to the ethical guidelines outlined in the Declaration of Helsinki, multivariate analysis assessed the prognostic value of p16.

Results: Our analysis demonstrated a significant correlation between HPV status and p16 expression in HNSCC cases. A vast majority of 58 (96.7%) HPV-+ cases exhibited p16 overexpression, contrasting sharply with only two (5%) in the HPV-- group. Patients with tumors that were both p16+ and HPV+ exhibited more favorable overall survival rates. In contrast, those with p16- and HPV- tumors experienced the poorest survival outcomes. Notably, having a p16-- status in HPV+ cases emerged as an independent factor for reduced survival. Additionally, the study revealed distinct variations in p16 expression based on tumor location, particularly within the oropharyngeal area.

Conclusion: The study established that p16 is a dependable indication for the existence of HPV in HNSCC and highlights its significant role as a prognostic factor, particularly in cases that are p16-- yet HPV-+. These findings underscore the importance of adopting site-specific treatment approaches in HNSCC management and contribute to a deeper understanding of p16's role in the disease, thereby aiding in more effective risk assessment and treatment planning.

## Introduction

Head and neck squamous cell carcinoma (HNSCC) poses a significant global health challenge, affecting various regions of the upper aerodigestive tract. With approximately 890,000 new cases diagnosed annually worldwide, HNSCC represents a major health risk to the general population [1]. Historically, alcohol and tobacco were the primary risk factors for HNSCC, but there has been a notable epidemiological shift due to the increasing prevalence of high-risk human papillomavirus (HPV) infections [2].

HPV-positive and HPV-negative HNSCC are distinct entities, each characterized by unique risk factors, molecular profiles, and clinical outcomes. Patients with HPV-positive HNSCC tend to be younger, with the disease more commonly occurring in the oropharyngeal region, and generally have a better prognosis [3]. Biomarkers such as p16 *CDKN2A* (cyclin-dependent kinase inhibitor-2A) have gained significant attention for their role in the early diagnosis and treatment of HNSCC. HPV disrupts the retinoblastoma protein (pRb) pathway, leading to the frequent overexpression of the cell cycle regulator and tumor suppressor p16 in HPV-positive HNSCC [4,5].

The expression of p16 is driven by the functional inactivation of pRb by the HPV E7 protein. The HPV oncogenes E6 and E7 play crucial roles in HPV-associated carcinogenesis by disrupting the functions of the tumor suppressors p53 and pRb, respectively. Unlike tobacco-induced HNSCC, which often involves *TP53* mutations, HPV-positive HNSCC typically retains wild-type *TP53*. The inactivation of Rb by E7 results in the upregulation of p16, which characterizes HPV-positive tumors by high p16 expression. Since E7 transcription is necessary for p16 upregulation, p16 overexpression is a reliable indicator of HPV involvement in carcinogenesis, making it a valuable biomarker for HPV-positive tumors [6,7].

This study aims to comprehensively investigate the role of p16 in both HPV-positive and HPV-negative HNSCC, focusing on its regulatory mechanisms, diagnostic and prognostic implications, and potential as a therapeutic target.

## Materials and methods

In this retrospective study conducted at a tertiary care facility, a cohort of 100 patients diagnosed with HNSCC was examined. The study focused on gathering information regarding the tumor's location, HPV status, p16 expression levels, and demographic characteristics of the patients. The sample size was estimated using the formula n=Z^2^×p×(1−p)/E^2^​, where n = sample size, Z = Z-score corresponding to the desired level of confidence (e.g., 1.96 for a 95% confidence level), p = estimated proportion of patients with the characteristic or outcome of interest in the population, and E = desired margin of error (precision).

The inclusion criteria include patients diagnosed with HNSCC, tissue samples collected from primary HNSCC tumors, and patients treated at the tertiary care facility where the study was conducted. The exclusion criteria include patients with incomplete medical records or missing data regarding tumor location, HPV status, or p16 expression levels; patients who received prior treatment for head and neck cancer, such as surgery, chemotherapy, or radiotherapy; patients with concurrent malignancies or significant medical comorbidities that could affect study outcomes; and patients with primary tumors located outside the head and neck region.

Tissue samples were collected from primary HNSCC tumors, which were then embedded in paraffin and preserved in formalin. HPV status was assessed using polymerase chain reaction (PCR), with a particular emphasis on identifying high-risk HPV strains. Additionally, p16 expression levels were evaluated through immunohistochemistry techniques.

A statistical analysis was performed to correlate p16 expression and HPV status with patient outcomes. Epi Info version 7 facilitated this analysis by enabling multivariate regression and survival analysis, allowing researchers to determine the predictive significance of p16 and HPV status in patient outcomes.

Ethical standards were strictly adhered to throughout the study. Approval was obtained from the Institutional Review Board with reference number IRB/SIMS/03/25314/2023, ensuring compliance with the ethical principles outlined in the Declaration of Helsinki. While informed consent is typically required in medical research, it was waived in this study due to its retrospective nature.

Statistical significance was established at a p-value of less than 0.05. The data were presented using odds ratios (OR), hazard ratios (HR), and confidence intervals (CI), providing a comprehensive understanding of the relationship between p16 expression, HPV status, and patient outcomes.

## Results

In our study involving 100 HNSCC patients, we explored the significance of p16 expression and its correlation with HPV status. The patients in our sample ranged in age from 58.7 years on average to 12.4 years on average. The majority of the patients were male, comprising 70 (70%) males and 30 (30%) females. The oro-pharynx accounted for 40 (40%) of instances, while the larynx and oral cavity each accounted for 30 (30%) of cases. The distribution of tumors in the head and neck region was not uniform (Table 1).

**Table 1:** Distribution of clinicopathological and sociodemographic features in patients SD: standard deviation; HPV: human papillomavirus

Characteristic	Total Patients (n=100)	HPV+ (n=60)	HPV- (n=40)
Age (in years), mean ±SD	58.7 ± 12.4	55.3 ± 11.2	63.9 ± 13.5
Gender (male), n (%)	70 (70%)	42 (70%)	28 (70%)
Female n (%)	30 (30%)	18 (30%)	12 (30%)
Tumor site n (%)			
Oropharynx	40 (40%)	36 (60%)	4 (10%)
Larynx	30 (30%)	10 (16.6%)	20 (50%)
Oral cavity	30 (30%)	14 (23.3%)	16(40%)
p16 expressions n (%)			
+	60 (60%)	58 (96.6%)	2 (5%)
-	40 (40%)	2 (3.33%)	38 (95%)

**Figure 1:**
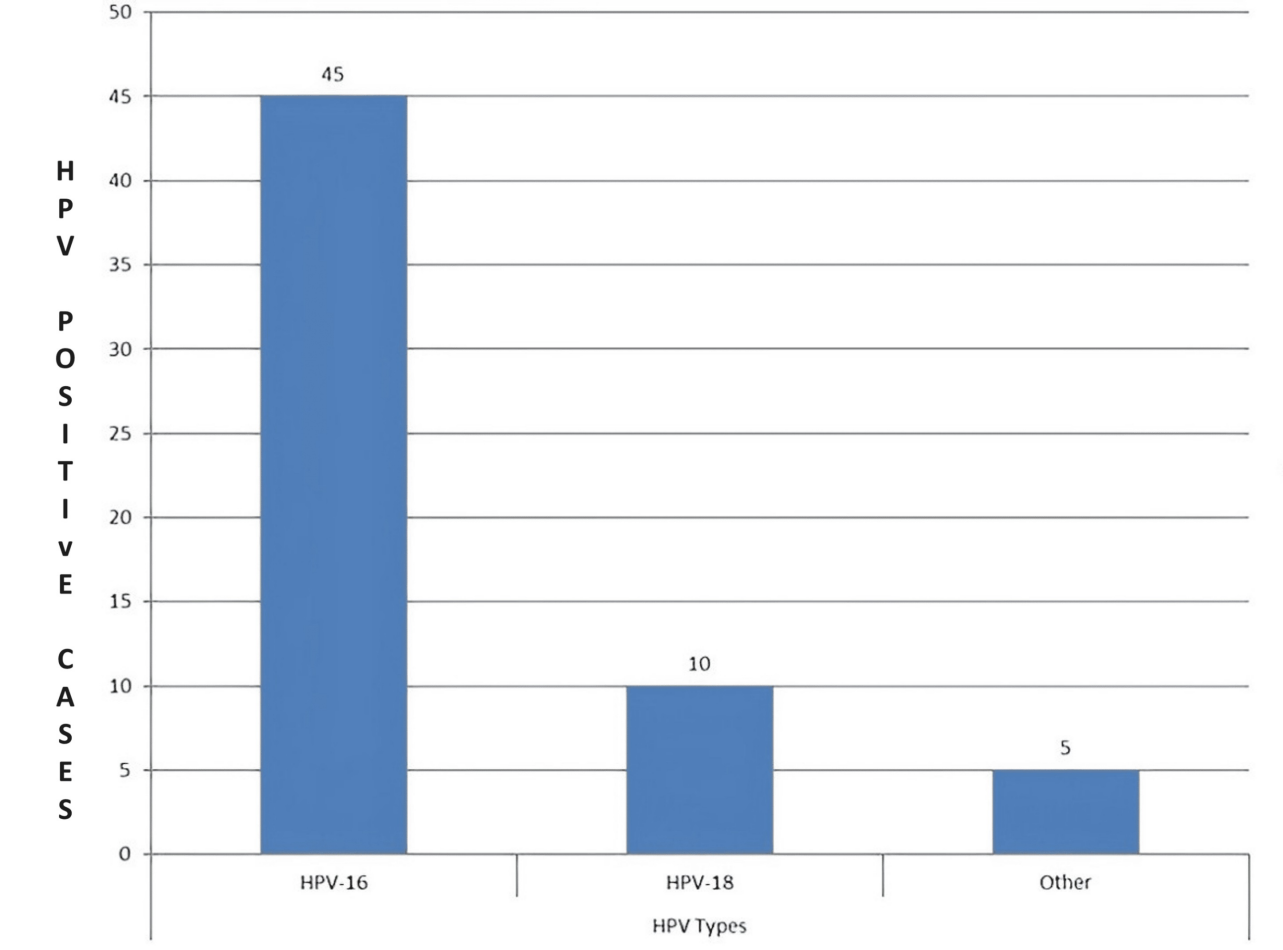
Elevated-risk HPV type distribution in HPV-positive HNSCC cases Y axis: number of HPV-positive cases HPV: human papillomavirus; HNSCC: head and neck squamous cell carcinoma

In our findings, 36 (60%) of the patients demonstrated p16 positivity. Importantly, there was a noticeable association between the presence of p16 and HPV-positive cases. This finding emphasizes the significance of p16 as a putative biomarker for HNSCC, especially in instances where HPV infection is present. Within our group of 60 HPV-positive patients, HPV-16 was found in 45 (75%) of the cases, making it the most common kind. The next most common was HPV-18, accounting for 10 (16.7%) of the cases. The remaining five (8.3%) cases were linked to other high-risk HPV strains. The several HPV varieties that contribute to HPV+HNSCC are highlighted in this data (Figure 1).

**Figure 2:**
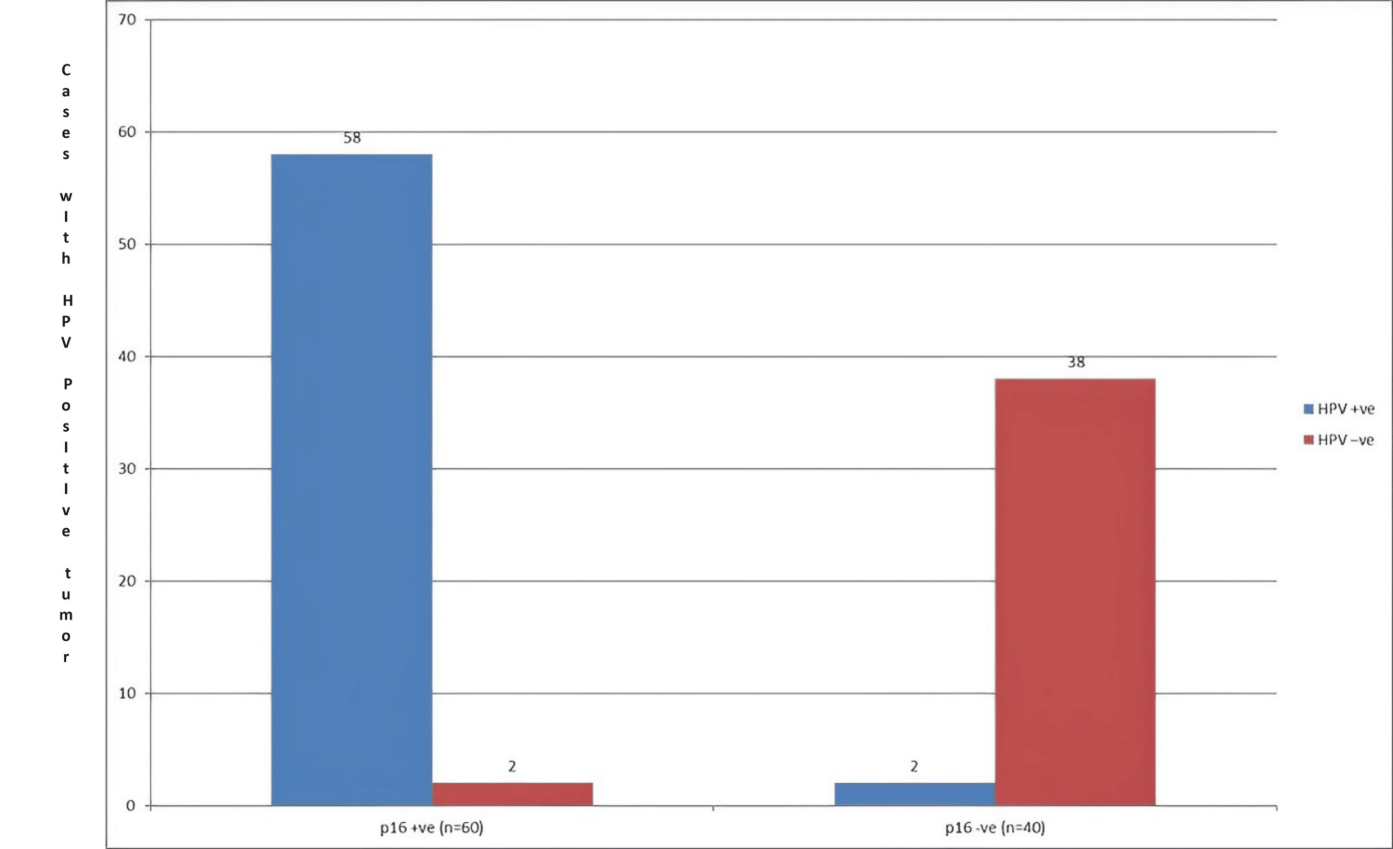
HPV status and p16 expression correspondence Y axis: number of cases with HPV-positive tumor HPV: human papillomavirus

The 58 (96%) patients with HPV-positive tumors had high levels of p16 expression. This result highlights the close relationship between HPV infection and p16 upregulation. On the other hand, p16 expression was detected in just two (4%) individuals with HPV-positive tumors. The important function of p16 as an efficient biomarker for the detection of HPV-positive HNSCC cases was underlined by the notable difference in p16 expression between the HPV-positive and negative groups (Figure 2).

Table 2 examines the impact of both HPV status and combination p16 expression on HNSCC patient survival. The results revealed that patients whose tumors were both p16-positive and HPV-positive had the most advantageous survival outcomes, with an average overall survival period of 72.5 months. In contrast, patients with tumors that were both p16-negative and HPV-negative faced a less favorable prognosis, as evidenced by a shorter average overall survival time of 43.9 months. These data underscore the pivotal role of p16 expression in conjunction with HPV status as key determinants in the survival rates of individuals diagnosed with HNSCC.

**Table 2:** Clinical results based on HPV status and p16 expression HPV: human papillomavirus; CI: confidence interval

Variable	p16+ or HPV+	p16- or HPV+	p16+ or HPV-	p16- or HPV-
Overall survival (in months) (95% CI)	72.5 (65.2-79.8)	52.1 (46.3-57.8)	58.7 (52.6-64.8)	43.9 (37.8-49.9)
Disease-free survival (in months) (95% CI)	65.2 (58.3-72.1)	42.6 (36.7-48.4)	51.8 (45.9-57.6)	35.4 (29.6-41.1)

Table 3 presented results from a multivariate analysis that assessed various factors affecting the overall survival rates in patients with HNSCC.

**Table 3:** Analysis with multiple variables for components affecting the collective survival HPV: human papillomavirus; CI: confidence interval

Variable	Hazard Ratio (HR)	95% CI	p-value
p16-+ or HPV-+	Reference		
p16-- or HPV-+	2.14	(1.62-2.83)	<0.001
p16-+ or HPV--	1.78	(1.32-2.40)	0.001
p16-- or HPV--	2.87	(2.14-3.84)	<0.001
Age (continuous)	1.04	(1.01-1.07)	0.012
Tumor stages (I/II/III/IV)	1.42	(1.20-1.67)	<0.001

This comprehensive analysis included variables such as age and tumor stage. The results revealed that in HPV-positive HNSCC cases, p16-negative patients had an HR of 2.14 (95% CI: 1.62-2.83) for tumors, significantly higher than the reference group of p16-positive or HPV-positive tumors after adjusting for age and tumor stage. This suggests that being p16-negative in the context of HPV positivity is an independent predictor of reduced overall survival. Additionally, the evaluation identified age and advanced tumor stage as critical factors influencing long-term patient outcomes.

**Figure 3:**
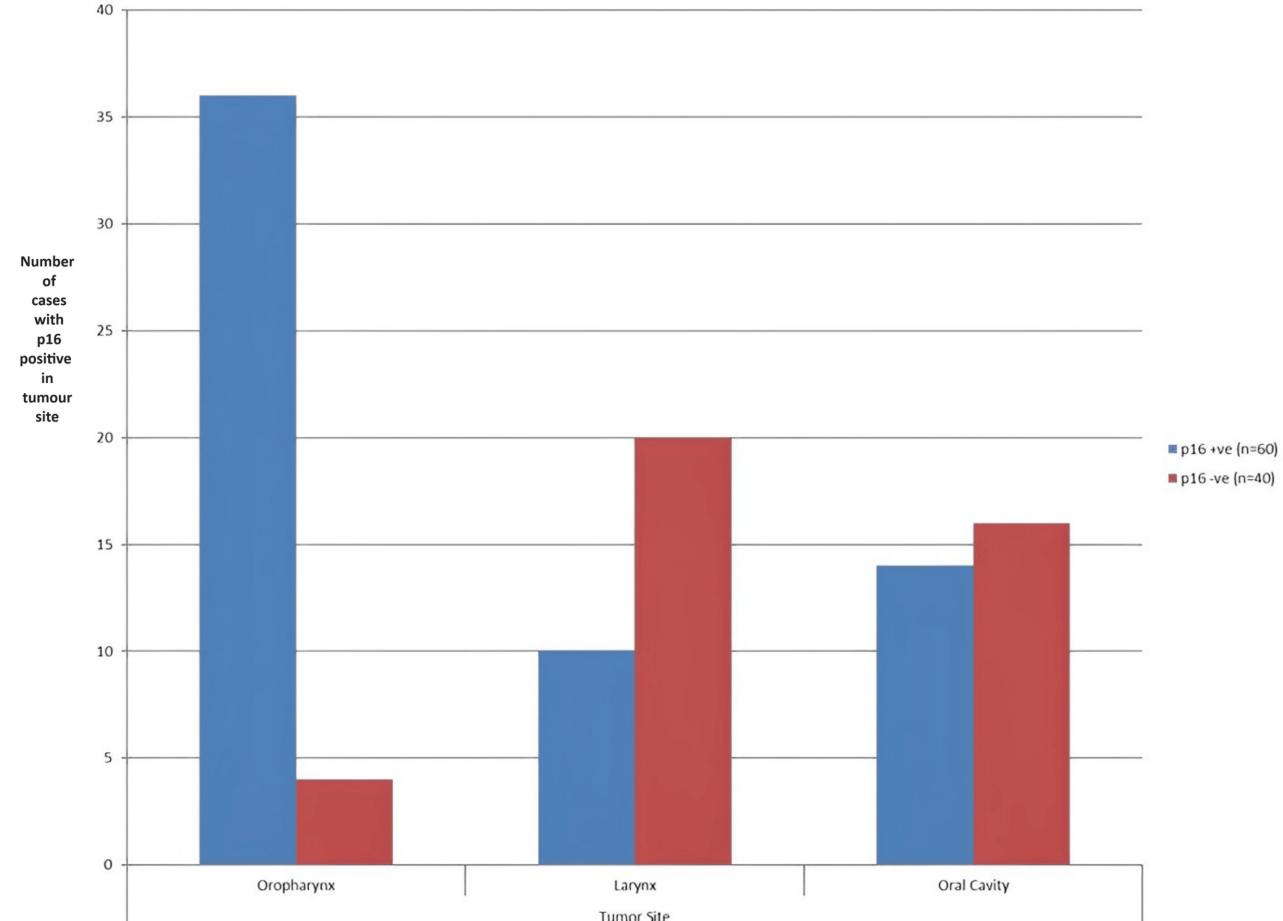
Expression concerning p16 in relation to the tumor site distribution Y axis: number of cases with p16 positivity at the tumor site

The oro-pharyngeal region was shown to have the greatest proportion of p16-positive patients, accounting for 36 (60%) of all p16-positive cases in the course of the study. The oral cavity, which accounted for 14 (23.3%) of the cases, had a moderate level of p16 expression, but the larynx had a significantly lower proportion of 10 (16.7%) of p16-positive cases. The p16 expression levels in the head and neck region vary, according to these data, which suggests that tumor-site-specific treatment strategies may be possible (Figure 3).

## Discussion

HNSCC is a complex disease influenced by various factors, including an elevated risk of HPV infection and p16 expression. This discussion integrates our study’s findings with existing literature, providing a deeper insight into the role of p16 in HNSCC.

Our results are in agreement with studies by Gillison et al. [5], O'Rorke et al. [8], and Dayyani et al. [9], confirming the established relationship between p16 expression and HPV status in HNSCC [5]. In our cohort, a significant majority (96.7%) of HPV-positive cases showed high levels of p16 expression. This is consistent with the mechanism where the HPV E7 oncoprotein disrupts the pRb pathway, leading to increased p16 expression. In contrast, only 5% of HPV-negative cases exhibited low p16 expression, similar to Lassen et al.’s findings [6,10-12]. These results underscore the validity of p16 as a surrogate marker for HPV in HNSCC.

Furthermore, our research highlights the prognostic importance of p16 in addition to HPV status. We observed significantly improved overall survival in patients with p16-positive or HPV-positive tumors, a trend reported by Ang et al. [3], Schroeder et al. [11], and Ndiaye et al. [12]. Conversely, p16-negative or HPV-negative tumors were associated with poorer survival rates, indicating a more aggressive disease course, as mentioned by Lassen et al. [6] and Gillison et al. [13]. This distinct survival pattern underscores the clinical significance of these biomarkers in assessing patient risk and guiding therapy.

Our findings are consistent with Weinberger et al. [7] and Mena et al. [14], indicating that p16-negative status in HPV-positive patients is an independent predictor of worse outcomes [7]. This highlights a particularly aggressive subgroup within HNSCC that is HPV-positive. Additionally, we noted variations in p16 expression across different HNSCC sites. The oropharynx exhibited the highest incidence of p16 positivity, consistent with studies by Ang et al. [3] and Dhull et al. [15], while p16-positive rates were lower in the oral cavity and larynx. According to Huang et al. [16], Bryant et al. [17], and Vitzthum and Mell [18], these site-specific variations underscore the heterogeneous nature of HNSCC and the necessity for site-specific therapeutic strategies.

Our findings have significant implications for clinical practice. Utilizing p16 as a biomarker is crucial for identifying high-risk patients, particularly within the HPV-positive group, thereby facilitating personalized treatment strategies. Additionally, recognizing site-specific differences in p16 expression can aid in developing tailored treatment plans, potentially leading to improved patient outcomes.

However, the retrospective nature of our study and its reliance on data from a single institution may limit the generalizability of our findings. The sample size in certain subgroups might also restrict our ability to detect subtle differences. Beyond emphasizing the prognostic significance of p16 and HPV status, our study highlights the need for further research into the molecular mechanisms driving the aggressive behavior of p16-negative/HPV-positive HNSCC. Understanding these pathways and genetic alterations could lead to targeted therapies that improve patient outcomes.

Moreover, integrating p16 and HPV testing into routine clinical practice could enhance diagnostic precision and prognostication. Identifying patients at higher risk of poor outcomes enables clinicians to tailor treatment regimens more effectively, potentially incorporating novel therapeutic agents that target the molecular aberrations present in these tumors. Future multicenter studies with larger and more diverse cohorts are essential to validate these findings and expand their applicability across different populations and healthcare settings.

Exploring the potential of combining p16 and HPV status with other emerging biomarkers may also provide a more comprehensive risk assessment and treatment framework. Advanced genomic and proteomic analyses could reveal additional molecular targets, paving the way for more sophisticated and personalized therapeutic interventions. Collaboration between research institutions and clinical centers will be vital in translating these findings into clinical practice, ultimately improving the prognosis and quality of life for patients with HNSCC.

## Conclusions

In conclusion, our study underscores the significance of p16 as a valuable biomarker in HNSCC, particularly in the context of HPV infection. The strong correlation observed between HPV status and p16 expression highlights the potential for p16 to serve as a prognostic indicator, aiding in risk stratification and treatment planning. Furthermore, the identification of distinct variations in p16 expression across different tumor locations emphasizes the need for personalized approaches to HNSCC management tailored to the specific biological characteristics of each patient's tumor.
